# Atypical Kawasaki disease presenting with macroscopic hematuria in an infant: a case report

**DOI:** 10.1186/s13256-022-03739-3

**Published:** 2023-01-11

**Authors:** V. Thadchanamoorthy, Kavinda Dayasiri, I. R. Ragunathan

**Affiliations:** 1 Faculty of Health Care Sciences, Eastern University, Chenkalady, Sri Lanka; 2 Faculty of Medicine, University of Kelaniya, Colombo, Sri Lanka; 3 Teaching Hospital, Batticaloa, Sri Lanka

**Keywords:** Atypical KD, Maculopapular rash, Prolonged fever

## Abstract

**Background:**

Kawasaki disease is an acute febrile condition in children. It affects mainly children under 5 years old, and is known to cause coronary artery abnormalities if treatment is delayed. The diagnosis rests mainly on clinical criteria. However, it is also known that some infants do not have diagnostic criteria sufficient enough for the diagnosis of Kawasaki disease. Further, children may rarely present with unusual features, and this entity is recognized as “Atypical Kawasaki disease.”

**Case presentation:**

We present the case of a 9-month-old Tamil boy who presented with sterile gross hematuria in association with prolonged fever, lymphadenopathy, and generalized maculopapular rash. He had high inflammatory markers and echocardiogram disclosed left coronary artery dilatation. The diagnosis of incomplete Kawasaki disease was confirmed based on clinical grounds supported by investigations and exclusion of differential diagnosis. The child showed a good response to intravenous immunoglobulin and aspirin.

**Conclusion:**

Kawasaki disease is one of the important differential diagnoses of protracted fever of unknown origin in very young children. Since delayed treatment is associated with a high risk of complications, atypical Kawasaki disease needs to be suspected in children presenting with unusual features such as macroscopic hematuria that occurs in association with unexplained prolonged fever.

## Background

Kawasaki disease (KD) is the most common cause of acquired heart disease in children in developed countries, and is an acute inflammatory vasculitis of unknown origin [1]. The disease affects small and medium-sized arteries, and can cause coronary artery weakening, aneurysm development, and myocardial infarction [2]. Atypical cases account for 15–20% of all patients with KD, and are observed mostly in children younger than 6 months or older than 5 years [3]. Atypical KD is also associated with a higher incidence of coronary artery abnormalities [3]. In the absence of specific tests, the diagnosis depends on the identification of diagnostic clinical features and the exclusion of other clinically similar conditions. Herein, we report an infant who presented with prolonged fever and macroscopic hematuria, and was diagnosed to have incomplete KD. The child responded to conventional treatment of Kawasaki disease satisfactorily.

## Case presentation

**Fig. 1:**
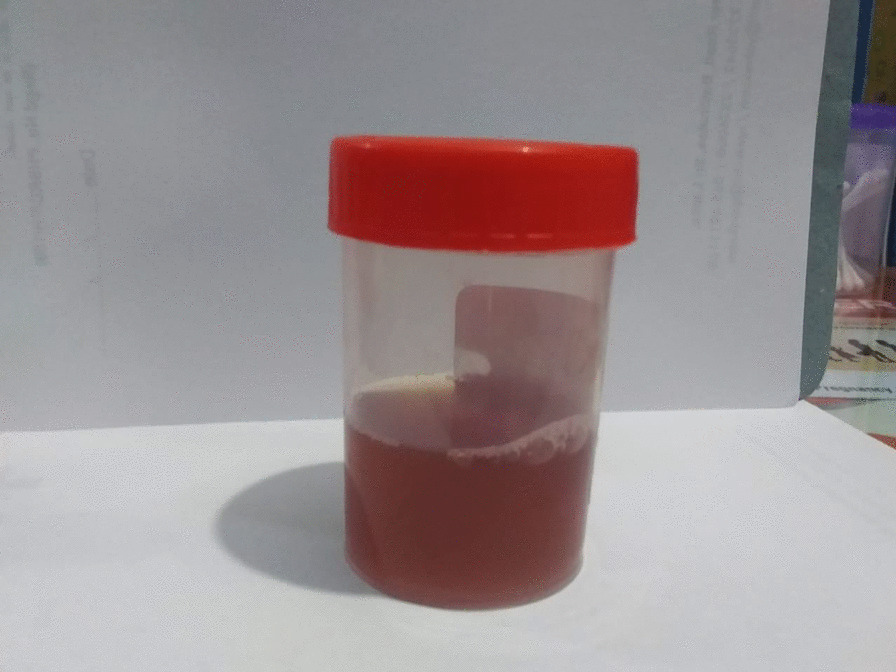
Macroscopic hematuria

A 9-month-old previously healthy Tamil boy was transferred from a local hospital for further evaluation of fever for 8 days and macroscopic hematuria (Fig. 1). The infant had diarrhoea during the initial phase of febrile illness and it subsided spontaneously. He showed macroscopic hematuria and pyuria, but his urine culture was negative. An antibiotic had also been given empirically for a urinary tract infection before he was transferred from the local hospital. However, fever did not respond to the antibiotic. His food intake was significantly reduced. He was crying most of the time. His immunization and development had been normal.

On examination, the baby was febrile (103 ˚F), ill, and irritable, and hydration was satisfactory. There was lymphadenopathy in the right cervical region which was 1.5 cm in size and a maculopapular rash was noted all over the body. There was no Bacillus Calmette–Guérin (BCG) reaction. Other systems examinations were unremarkable.

**Fig. 2:**
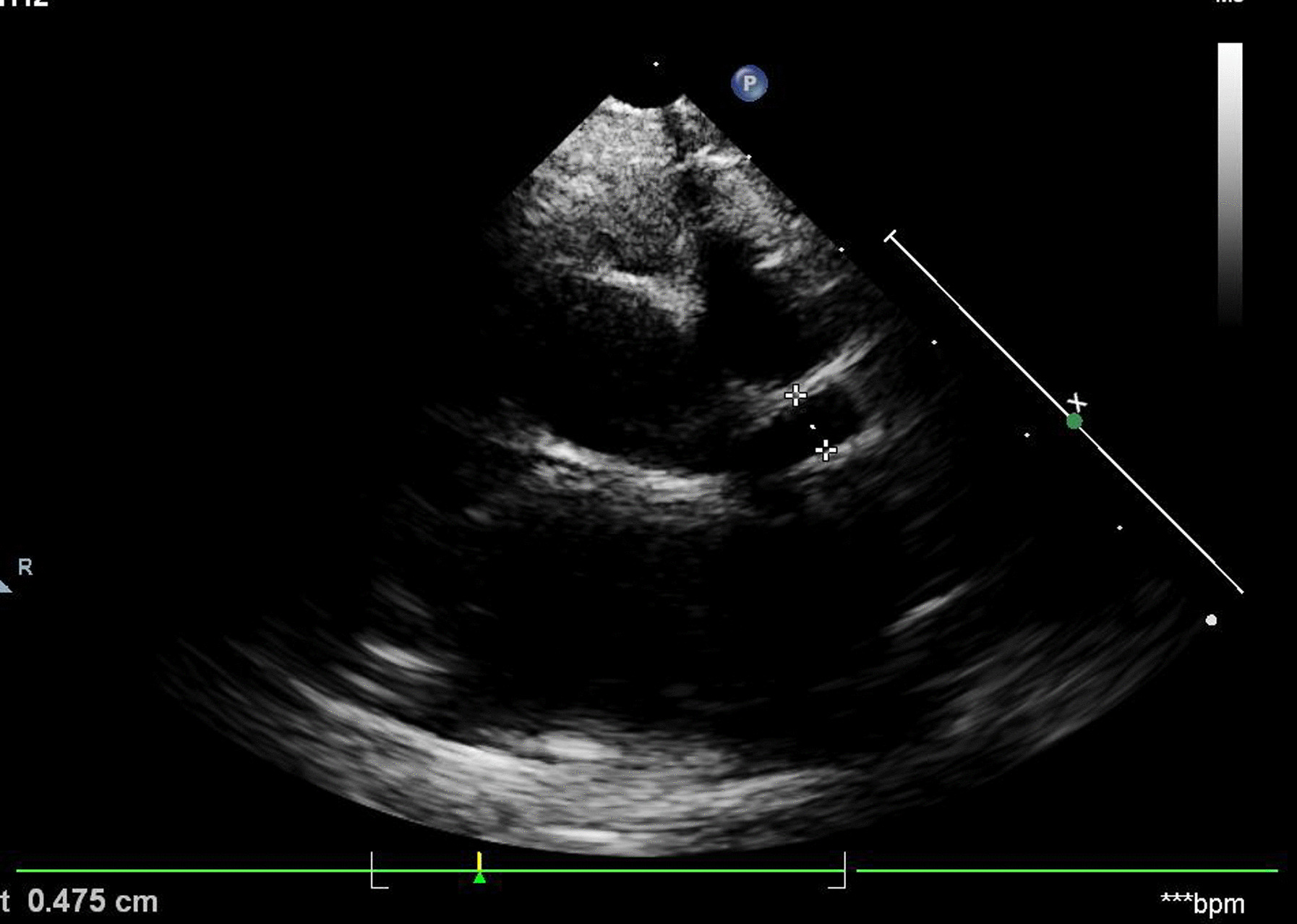
Dilatation of left coronary artery

Urine microscopy on several occasions revealed hematuria and pyuria. Blood investigations showed high white blood count (24 × 10^3 ^/cumm, neutrophils 80%), low hemoglobin (8 g/dL), slightly high platelets (440 × 10^3^/cumm), high C-reactive protein (96 mg/dL), high erythrocyte sedimentation rate (ESR, 80 mm/1^st^ hour), high liver functions [alanine transaminase (ALT) 98 IU/dL, aspartate transaminase (AST) 120 IU/dL, gamma-glutamyl transferase (GGT) 156 IU/dL] and low serum protein (total 5.8 mg/dL, albumin 2.4 mg/dL). Renal function, serum ferritin, and lipid profile were within normal limits. The cerebrospinal fluid analysis revealed normal results. Results of serology for Epstein–Barr virus, cytomegalovirus, influenza, and mycoplasma were within normal limits. Urine, cerebrospinal fluid (CSF), and blood cultures were sterile. The chest x-ray had been normal. Ultrasound abdomen showed mild hepatomegaly with the normal size gall bladder. The echocardiogram (ECHO) showed left coronary artery dilatation (4.75 mm) on day 12 of illness (Fig. 2).

He was diagnosed to have atypical Kawasaki disease and started on conventional high-dose intravenous immunoglobulin (IVIG) (2 g/kg) and high-dose aspirin (100 mg/kg). He responded very well, within 24 hours of IVIG. Aspirin was continued until ESR became normal and changed to low-dose aspirin subsequently (5 mg/kg). The second ECHO after 2 weeks of treatment showed persistent dilatation of the left coronary artery (3.5 mm). He was discharged after 3 weeks of illness with low dose aspirin for 6 weeks and follow-up in clinic. At 6 weeks, all inflammatory markers had become normal; however, he had 3 mm dilatation in the left coronary artery, and he was recommended for long-term follow-up and low-dose aspirin therapy. He was reviewed monthly with aspirin and repeated ECHO at 1 year showed normal findings. After 2 years of follow-up in tertiary care hospital, he was referred to a local clinic for routine follow-up and immunization.

## Discussion

KD is an autoimmune vasculitis of unidentified factors. It mainly affects young children aged between 3 months and 10 years. Kawasaki disease was first reported in 1961 [3]. The incidence of KD in Northeast Asian countries including Japan, South Korea, China, and Taiwan is 10–30 times higher than in the USA and Europe [6]. The male-to-female ratio is approximately 1.5:1. Peak incidence is noted between January and March, suggesting an environmental contribution [7]. Our child was a 9-month-old male.

There are no specific diagnostic investigations to differentiate typical and atypical Kawasaki disease. Typical KD should have essential criteria: evidence of prolonged fever (≥ 5 days) associated with the presence of at least four out of five principal clinical features (change in extremities, polymorphous exanthema, bilateral bulbar conjunctival injection without exudate, changes in the lips and oral cavity, cervical lymphadenopathy of more than 1.5 cm) [8]. All the clinical criteria may not be present initially, but evolve subsequently in the second week. An incomplete presentation might have an unexplained fever for ≥ 5 days associated with two or three of the principal clinical features described above [9]. Our patient had fever for more than 5 days, with maculopapular rash and cervical lymphadenopathy. Although not diagnostic, there are some less common features, including gastrointestinal (diarrhoea, emesis, and abdominal pain), respiratory (cough and rhinorrhea), rheumatologic (joint pain and swelling) symptoms [7], and genitourinary symptoms such as urethritis associated with sterile hematuria and pyuria. Diarrhoea and genitourinary symptoms were the presenting features of the reported child. The laboratory investigations (for example, elevated erythrocyte sedimentation rate and C-reactive protein level, hyponatremia, hypoalbuminemia) and echocardiographic findings might support the diagnosis [7]. About 15–25% of untreated patients might develop coronary artery aneurysms or ectasia and also end up in myocardial infarction, sudden death, or ischemic heart disease[4]. When the initial echocardiography showed no coronary artery alterations, repeat ECHO is mandatory in all patients to confirm coronary abnormalities after 10 days of febrile illness. The reported child had coronary artery dilatation on day 12 of febrile illness.

Diagnosis of incomplete KD depends on incomplete criteria with echocardiographic (ECHO) features with or without elevated inflammatory markers, and exclusion of other similar diseases such as drug hypersensitivity, juvenile idiopathic arthritis, staphylococcal scaled skin syndrome, Stevens–Johnson syndrome, streptococcal scarlet skin syndrome, toxic shock syndrome, and viral infection [7]. Our child had high CRP, ESR, and liver function, high white blood count (WBC) with neutrophil predominant, low hemoglobin, high platelets, and macroscopic hematuria.

According to the American Heart Association (AHA) guidelines, intravenous immunoglobulin (IVIG) and high-dose aspirin have been the main mode of treatment in Kawasaki management [7, 10, 11]. Although the action of IVIG is not known, it is said that it has some immunomodulatory effects such as cytokine production, influence on T-cell activity, and suppression of antibody synthesis [7]. A single dose of 2 g per kg is administered within 10 days of illness, or later if the patient has persistent fever or aneurysms, or persistent inflammation supported by markers. It is estimated that the development of coronary artery abnormalities has been reduced from 25% to 5% and the formation of giant aneurysms to 1%. Besides, the acute disease benefits from high-dose aspirin initially (80–100 mg/kg) to reduce inflammation. Once inflammatory markers return to normal, low-dose aspirin (3–5 mg/kg) as a single dose is suggested to reduce platelet activation and prevent thrombosis for 6–8 weeks. Aspirin should be continued indefinitely if coronary abnormalities persist on follow-up ECHO [10, 11]. It is recommended to give steroids in refractory cases; however, this practice is controversial. Although the described case responded symptomatically to aspirin and IVIG, follow-up ECHO showed persistence of coronary abnormalities. He was scheduled for long-term follow-up with the continuation of aspirin. He was reviewed regularly at pediatric and cardiology clinics. The 2D echocardiogram repeated at 1 year showed normal findings. After 2 years of follow-up in a tertiary care hospital, he was referred to the local hospital for routine follow-up and immunization. As the patient had a risk of Reye syndrome with influenza and varicella, his parents were informed regarding the need for vaccination against those infections [4, 7].

## Conclusions

Kawasaki is one of the important differential diagnoses of protracted fever of unknown origin and sterile hematuria in very young children. A high index of suspicion is crucial since delayed treatment is associated with a high risk of complications. It is important that clinicians re-examine children to detect the new physical signs arising in the subsequent weeks when there is a suspicion of Kawasaki disease. Moreover, negative echocardiogram in the early days of illness will not exclude Kawasaki disease, as coronary changes occur in the second week of illness. Early diagnosis and treatment might prevent the development of coronary artery disease.

## Data Availability

The data that support the findings of this case report are available from Medical Records Department, Batticaloa Teaching Hospital, but restrictions apply to the availability of these data, which were used under license for the current report and so are not publicly available. Data are, however, available from the authors upon reasonable request and with permission of Medical Records Department, Batticaloa Teaching Hospital, Sri Lanka.

## Abbreviations

KD: Kawasaki disease
ECHO: Echocardiogram
IVIG: Intravenous immunoglobulin
